# Osmotic Pressure and Diffusion of Ions in Charged Nanopores

**DOI:** 10.1021/acs.langmuir.1c02267

**Published:** 2021-11-25

**Authors:** P. Apel, M. Bondarenko, Yu. Yamauchi, A. Yaroshchuk

**Affiliations:** † Joint Institute for Nuclear Research, Joliot-Curie strasse 6, 141980 Dubna, Russian Federation; ‡ F. D. Ovcharenko Institute of Bio-Colloid Chemistry, National Academy of Sciences of Ukraine, Vernadskiy blvd. 42, 03142 Kyiv, Ukraine; § ICREA, pg. L. Companys 23, 08010 Barcelona, Spain; ∥ Department of Chemical Engineering, Universitat Politècnica de Catalunya, av. Diagonal 647, 08028 Barcelona, Spain

## Abstract

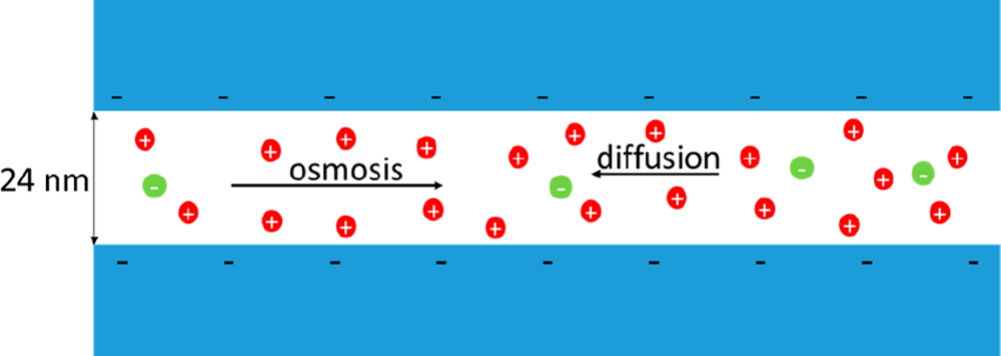

The transport of ions and water in nanopores is of interest for a number of natural and technological processes. Due to their practically identical long straight cylindrical pores, nanoporous track-etched membranes are suitable materials for investigation of its mechanisms. This communication reports on simultaneous measurements of osmotic pressure and salt diffusion with a 24 nm pore track-etched membrane. Due to the use of dilute electrolyte solutions (1–4 mM KCl and LiCl), this pore size was commensurate with the Debye screening length. Advanced interpretation of experimental results using a full version of the space-charge model has revealed that osmotic pressure and salt diffusion can be quantitatively correlated with electrostatic interactions of ions with charged nanopore walls. The surface-charge density is shown to increase with electrolyte concentration in agreement with the mechanism of deprotonation of weakly acidic surface groups. Moreover, a lack of significant surface-charge dependence on the kind of cation (K^+^ or Li^+^) demonstrates that binding of salt counterions does not play a major role in this system.

## Introduction

Charged nanoporous membranes show interesting ion-separation behavior controlled by fixed electrical charges on their pore surface.^1−3^ They also feature rather high efficiencies in electrokinetic energy conversion.^4−6^ Therefore, quantitative characterization of their electrochemical properties is of interest. Charged UF membranes (with nanoscale pore sizes) have been described in the literature, but the principal motivation for the studies has been practical considerations, in particular, an improved fouling resistance due to electrostatic repulsion of (usually negatively charged) natural macromolecules and colloids by an equally negatively charged membrane surface.^7−9^ However, to have sufficiently high water fluxes, practical charged UF membranes are asymmetric or composite. Such multilayer structure complicates considerably quantitative characterization of their nanoporous active layers.^10,11^ Nanoporous track-etched membranes^12^ are more amenable to quantitative characterization due to their monolayer structure. In addition, because of the practically identical straight cylindrical pores (narrow pore size distribution),^13^ they are suitable for a quantitative verification of ion- and solvent transport models.

Electrokinetic properties of track-etched membranes have often been characterized via measurements of streaming potential,^14−16^ although with nanoporous grades (due to a concomitant salt rejection) stationary voltage is actually a filtration potential.^17,18^ In this case, observation of genuine streaming potential is nontrivial and requires time-resolved measurements,^17,18^ which was not always recognized.^19^ Osmotic pressure and concomitant salt diffusion have also been studied;^20,21^ however, ref (20) was mostly theoretical and did not describe the experimental procedures, while ref (21) provided no mechanistic interpretation. Yaroshchuk et al.^22^ used measurements of transient membrane potential after current switch-off, while hydraulic permeability (and, thus, electro-viscosity) and pressure-driven salt rejection were studied experimentally in refs (19) and (23).

A space-charge model is rather well established^24−29^ and validated in relatively dilute electrolyte solutions just for the kind of nanopores investigated in this study.^17,22^ Peters et al.^25^ placed an emphasis on simplifications of repeated integrations, but below we will see that just two integrations are required, which is not a problem numerically. Hijnen et al.^20^ paid considerable attention to approximate analytical solutions of the Poisson–Boltzmann equation, but their use brings about unnecessary uncertainty concerning their applicability. Moreover, in the interpretation of experimental data, it is difficult to make any a priori assumptions concerning the magnitude of electrostatic potential (e.g., high potentials) often used in the approximate solutions of the Poisson–Boltzmann equation. Thus, for instance, in ref (30) considerable attention has been paid to the limits of applicability of the so-called uniform potential approximation, but it is not applicable to the nanopores of interest in this study. Studies^26−29^ used numerical solution of the Poisson–Boltzmann equation and numerical integrations, but this was applied to membrane phenomena other than those investigated in this work.

In this study, we will report on parallel measurements of osmotic pressure and diffusion with a nanoporous track-etched membrane in dilute KCl and LiCl solutions. These electrolytes were selected as simple common salts whose cations have most different diffusion coefficients. The purpose was demonstrating that the model captures the (nontrivial) dependence on the ion diffusion coefficients well. Innovative interpretation of the simultaneous measurements will make possible elimination of (poorly controllable) contribution of unstirred layers. The use of the full version of the space-charge model eliminates uncertainties related to the previous use of approximate solutions of the Poisson–Boltzmann equation for the interpretation of osmotic pressure and salt diffusion. We will confirm the mechanism of surface-charge formation due to dissociation of weakly acidic groups and additionally demonstrate that binding of cations of two studied salts (KCl and LiCl) does not have a noticeable impact for the studied membrane. The results of this work are important for an improved understanding of mechanisms of ion and water transport in nanopores and for optimization of applications of nanoporous charged membranes.

## Theory

In the Supporting Information, these equations are derived for the description of the one-dimensional zero-current volume and salt transfer across monolayer nanoporous membranes
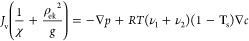
1
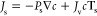
2where *J*_v_ is the transmembrane volume flow, χ is the membrane hydraulic permeability at zero voltage gradient (χ = γ*r*_p_^2^/8η for membranes with cylindrical pores, where γ is the membrane porosity, *r*_p_ is the pore radius, and η is the dynamic solution viscosity), *p* is the hydrostatic pressure in the virtual solution (see ref (31) and the Supporting Information for the definition), *c* is the salt concentration in the virtual solution, *J*_s_ is the salt flux, and ν_*i*_ terms are ion stoichiometric coefficients (*Z*_1_ν_1_ + *Z*_2_ν_2_ = 0, where *Z*_1_ and *Z*_2_ are ion charges)
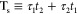
3is the salt transmission coefficient (1 – salt reflection coefficient), τ_*i*_ terms are the so-called ion transmission coefficients (defined below)
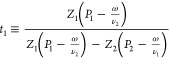
4is the transport number of ion “1” at zero transmembrane volume flow, *t*_2_ ≡ 1 – *t*_1_.
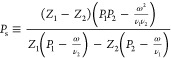
5is the salt diffusion permeability at zero transmembrane volume flow.

Equation 1 shows that the more easily measurable hydraulic permeability at zero electric current is equal to
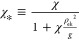
6where
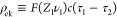
7is the electrokinetic charge density (the proportionality coefficient between electric-current density and volume flux under streaming-current conditions, i.e., ∇*c* = 0, ∇φ = 0)
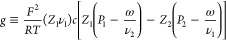
8is the membrane electric conductivity at zero transmembrane volume flow.

For capillary models, the coefficients in eqs 1–8 are defined by (see the Supporting Information)
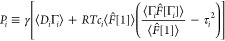
9
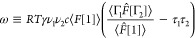
10
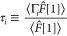
11where γ is the membrane porosity and τ*_i_* terms are the ion transmission coefficients quantifying the extent to which ions are convectively entrained by the volume flow.^a^ In principle, these coefficients can be affected by steric hindrance,^31^ but this is not significant in nanopores whose size is much larger than the ion size (the focus of this study). On the basis of the same considerations, we also neglect the effect of steric hindrance on ion diffusion and consider ion diffusion coefficients in nanopores constant and equal to those in a bulk electrolyte solution.

**Figure 1:**
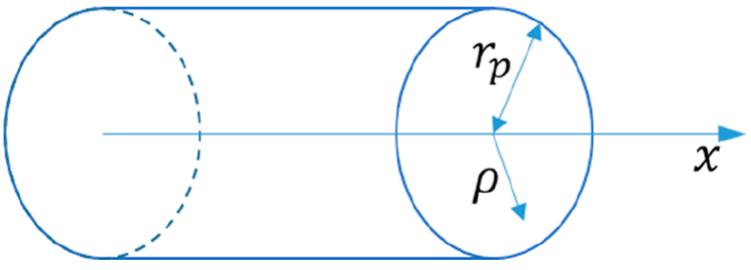
Model pore geometry.

The linear functional operator *F̂*[] gives the solution to this equation
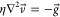
12where Γ*_i_* terms are the coefficients of ion partitioning between a given point inside the pore and virtual solution and the brackets, ⟨⟩, mean integration over the pore cross section and scaling on its area (*c*_*i*_ ≡ ν_*i*_*c*). For long straight capillaries away from their edges, only one velocity component (along the capillary) is non-zero. In “symmetrical” capillaries (e.g., cylindrical, slit-like), this component depends on only one coordinate, for example, radial position within cylindrical capillaries. In the case of the solvent, Γ_*i*_ = 1, and for *F̂*[1], we recover the classical Stokes equation and the well-known parabolic Hagen–Poiseuille velocity profile [in cylindrical capillaries (see Figure 1)]
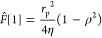
13where ρ is the dimensionless (scaled on the pore radius) radial coordinate inside the capillary. In “symmetrical” long straight capillaries, eq 12 can be easily solved in quadratures for an arbitrary right-hand side being a function of only one cross-sectional coordinate (e.g., radial position). For cylindrical pores,^32^ this results in
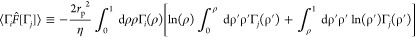
14
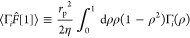
15
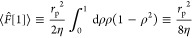
16The space-charge model postulates ion partitioning due to electrostatic interactions with fixed charges on the nanopore walls. The Poisson–Boltzmann (PB) equation for the quasi-equilibrium dimensionless electrostatic potential, ψ, describes these interactions
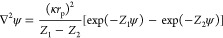
17where κ is the reciprocal Debye screening length defined as
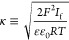
18
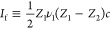
19is the ionic strength in the virtual solution. The boundary conditions are zero potential derivative at the capillary axis (from the symmetry) and a given electric-charge density (potential derivative) at the capillary wall.
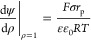
20where σ is the surface-charge density. One can also consider the so-called charge-regulation boundary condition,^33^ but in this study we will use the simpler condition of a constant charge density given by eq 20. The PB equation has several approximate solutions, but they are not applicable for the parameter combinations corresponding to the experiments reported in this study. Therefore, the PB equation was solved numerically. The repeated integrations featured in eqs 9, 10, 14, and 15 have been performed numerically, too. See the Supporting Information for more detail on the procedures.

In experiments, we measured osmotic pressure. This occurs at zero transmembrane volume flow. By integrating eq 2 over the transmembrane coordinate (at sufficiently small concentration differences), we can relate the transmembrane difference of hydrostatic pressures to the concentration difference in this simple way
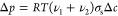
21where σ_*s*_ ≡ 1 – *Τ*_s_ is the salt reflection coefficient, which can be determined directly from the measured hydrostatic pressure difference (provided that the salt concentration difference is known or measured). The salt diffusion permeability was estimated from the rate of change of the salt concentration difference between the source and the receiving compartments (see below). In our experiments, the relative concentration difference changes were rather small, so the accuracy of salt flux estimates is not very high. Accordingly, it is difficult to differentiate between the rate of salt flux occurring during the initial stages of the experiment [where the osmotic flow is still non-zero (see Figure 3a)] and during the later stages where the salt diffusion occurs at practically zero transmembrane volume flow. Nonetheless, the theoretical model affords estimates of the possible contribution of volume transfer to salt diffusion [and demonstration that it is low to moderate (see below)]. While defining the diffusion permeability at non-zero volume flow, we should account for the concentration changes due to solvent transfer from the receiving compartment and consider the so-called salt chemical flux defined as
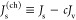
22and quantify the rate of salt concentration changes in the compartment receiving salt flux (and losing volume). In our experiments, the contribution of volume transfer to the salt diffusion is largest at the early stages where the transmembrane pressure difference is still very small, so for overestimates, we can assume it to be equal to zero. By setting ∇*p* = 0 in eq 1 and by substituting eqs 2 and 22, we find that the “initial” salt diffusion permeability (at zero hydrostatic pressure difference) is related to the “zero-flow” permeability this way.
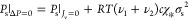
23Because all of the factors in the second term on the right-hand side of eq 23 are positive, due to volume transfer, salt diffusion permeability always increases.

## Contribution of Unstirred Layers

The membranes used in this study are very thin (∼10 μm). Their porosity is also relatively high (for this kind of membrane). As a result, their effective thickness (defined as the actual thickness divided by the porosity) is rather small (∼300 μm). Given often reported typical thicknesses of unstirred layers of approximately 50–100 μm in stirred test cells,^34^ it is difficult to ensure full membrane control especially in cells agitated by magnetic stirrers.^b^ The existence of external mass-transfer limitations causes a decrease in the concentration difference occurring on the membrane. Using the model of in-series connection of diffusion resistances, it is easy to show that the decrease in the concentration difference occurring on the membrane is equal to the ratio of the diffusion resistance of the membrane, *R*_m_, and of the in-series connection of the membrane and two unstirred layers (each having diffusion resistance *R*_ul_): *R*_m_/(*R*_m_ + 2*R*_ul_). This decreased concentration difference gives rise to a lower osmotic pressure and a smaller diffusion salt flux. Thus, interpretation of our measurements can be affected by unstirred layers whose thickness is quite difficult to determine, especially in stirred cells with magnetic stirrers located at the cell bottom (see below). Fortunately, at sufficiently small concentration differences, the osmotic pressure and diffusion salt flux are affected by unstirred layers to approximately the same extent (because they are controlled by the same reduced salt concentration difference). Therefore, the ratio of the salt reflection coefficient to the salt diffusion permeability is practically unaffected. This will be used below in the interpretation of experimental data.

## Experimental Section

### Membrane

**Figure 2:**
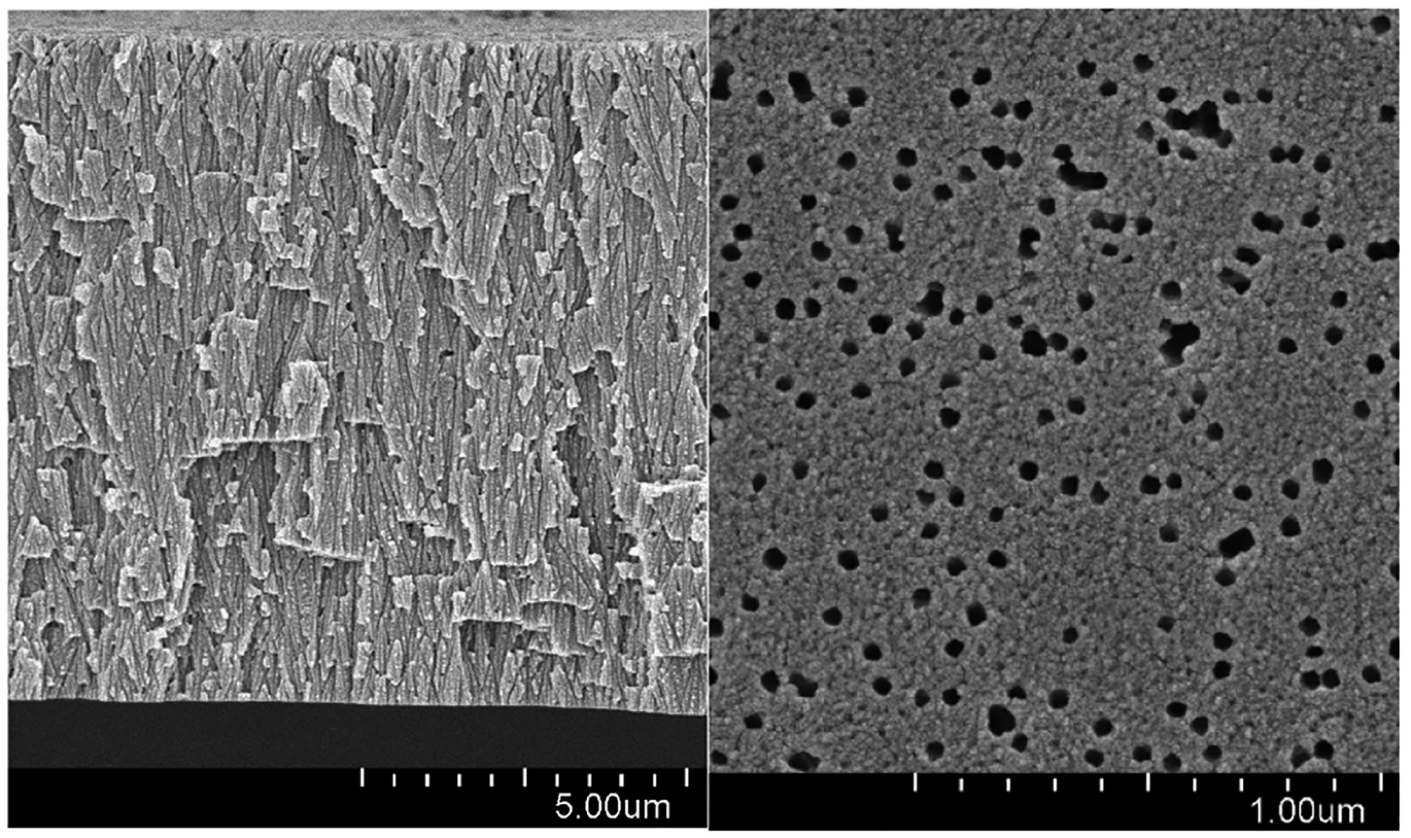
Structure of the membrane (SEM): cross section (left) and face (right).

A poly(ethylene terephthalate) (PET) track-etched membrane (TEM) was obtained by irradiating a PET film with accelerated Xe ions from the U-300 cyclotron at the Flerov Laboratory of Nuclear Reactions, Joint Institute for Nuclear Research, followed by a 3 h exposure to ultraviolet radiation (wavelength of >285 nm, intensity of 5 W m^–2^) and a subsequent chemical etching in a sodium hydroxide solution (0.56 M NaOH, 80 °C). The average surface density of pores (8 × 10^13^ m^–2^) was determined via scanning electron microscopy (SEM), with the total number of counts not less than 1000. The axes of the pore channels were uniformly distributed within a range of angles from −30° to 30° with respect to the normal [to reduce pore overlap along their whole length (see the Supporting Information for more details)], so the effective pore length was larger than the membrane thickness by a factor of 1.046. This was taken into account when estimating the effective pore diameter and the salt diffusivity reduction factor. Typical SEM images of the membrane are shown in Figure 2.

### Measurements of Osmotic Pressure and Salt Diffusion Flux

The experiments were carried out in a two-compartment transparent Plexiglas cell described previously.^21^ A sample with an exposed surface area of 3.14 cm^2^ was placed into a seat, having a diameter of 30 mm, in the partition between the two parts of the cell. The compartments (volumes of 300 and 250 mL) of the cell were filled with salt solutions of different concentrations. The higher-concentration compartment (300 mL) was equipped with a vertical polysulfone capillary having an internal diameter of 1.00 ± 0.02 mm. This compartment was stirred by a Teflon-coated magnetic stirrer. The solution in the other compartment (250 mL) was stirred by a propeller stirrer. After the cell had been assembled and filled and stirring had begun, the initial level of the liquid in the capillary was non-zero (due to capillary action). This did not influence the height of the maximum, which is of primary interest. Well-defined initial conditions would be important in an attempt to use the whole time dependence of hydrostatic pressure for the interpretation. However, this is complicated by membrane deformation, which is difficult to control. The height of the solution level in the capillary was measured as a function of time. The solutions were partially deaerated before use by being heated at 60 °C for 1 h followed by a further 2 min treatment in an ultrasonic bath. This prevented formation of air bubbles in the higher-concentration compartment and in the measuring capillary. The pH of the solutions was in the range of 5.9–6.1.

The specific electric conductivity in the lower-concentration compartment was monitored with a ProfLine Cond 3110 conductometer. The concentration in the other compartment was estimated from the material balances. The experiments were performed at a room temperature of 22 ± 1 °C. A nearly linear dependence of the electrical conductivity on the salt concentration was found under the experimental conditions (low concentrations of ≤4 mM). The coefficients used to calculate the concentrations from the specific conductivities were 68.3 and 46.8  for KCl and LiCl, respectively. Errors in concentration measurements were mainly caused by temperature fluctuations.

## Results and Discussion

### Numerical Estimates of Osmotic Correction to Diffusion Permeability

At non-zero transmembrane volume flows, the increased rate of salt concentration changes in the salt-receiving compartment is due to the (partial) salt rejection accompanying the osmotic flow, which leaves the compartment. Because both the rate of osmosis and the salt rejection are proportional to the salt reflection coefficient, the correction is quadratic in it. As we can see from eq 23, the osmotic correction to the membrane diffusion permeability can be noticeable just for the investigated “intermediate” nanoporous charged membranes because such membranes can have relatively large hydraulic permeabilities and not overly small salt reflection coefficients at not overly low salt concentrations. Nevertheless, the effect is still limited. For the parameter combinations corresponding to the membranes and conditions described in the Experimental Section, we have estimated the correction to be ≲15% for KCl and still lower for LiCl solutions (see the Supporting Information). Given the relatively low accuracy of determination of the rate of change of salt concentration (see above), we will neglect this relatively small correction and use the zero-flow diffusion permeability (eq 5) for the fitting of experimental data by the space-charge model.

### Experimental Data and Their Interpretation

**Figure 3:**
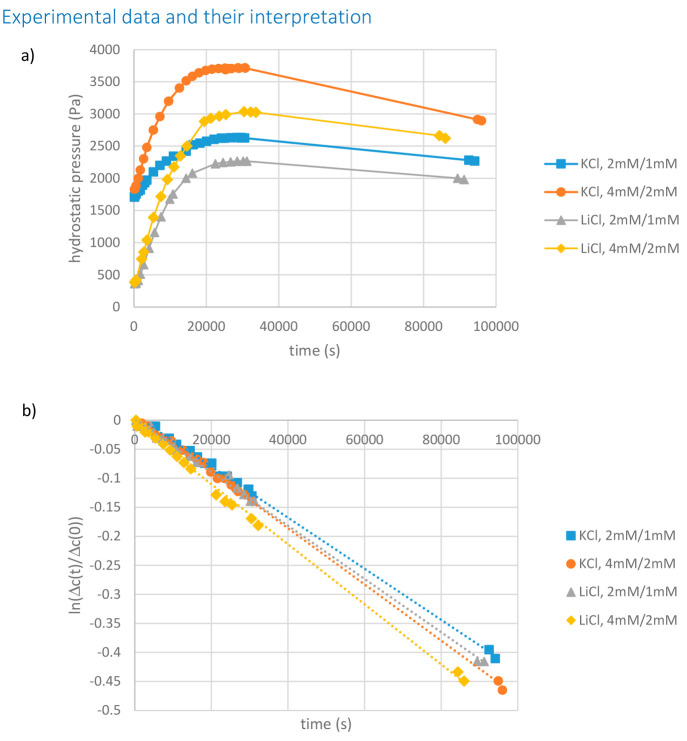
(a) Time dependencies of hydrostatic pressure in the closed compartment and (b) concentration difference between the compartments. The solid lines in panel a are guides for the eye. The dotted lines in panel b are linear approximations of experimental data whose slopes were used for the determination of membrane diffusion permeance according to eq 24.

Figure 3 shows experimental time dependencies of hydrostatic pressure in the hydraulically closed compartment and of salt concentration difference between the compartments (presented in the coordinates of eq 24). The hydrostatic pressure exhibits maxima. They occur because the salt concentration difference (the driving force of osmosis) gradually decreases with time. Initially, this is overcompensated by the buildup of hydrostatic pressure, but once the maximum (osmotic) pressure is approached, the effect of concentration difference reduction becomes visible. Around the maxima, the transmembrane volume flow is very small, so conditions of observation of osmotic pressure are fulfilled. Figure 3b shows that by the time of occurrence of the maxima the concentration difference between the compartments already somewhat decreases compared to the initial value. However, given that the salt concentrations in the compartments are continuously monitored, this is not a problem and the actual value of the concentration difference corresponding to the maximum hydrostatic pressure was used for the estimates of the salt reflection coefficient from eq 21.

The membrane diffusion permeance was estimated by using this classical relationship for the time dependence of diffusant concentration difference between compartments in a stirred two-compartment cell^34^
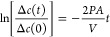
24where *P* is the sought-after permeance, *A* is the exposed membrane area, and *V* is the half-cell volume (assuming the volumes of both half-cells are equal).^c^ The membrane permeability to salt, *P*_s_, was obtained via multiplying the permeance, *P*, by the adjusted membrane thickness [*L* = 11.3 μm, corrected for the pore angle distribution (see the Experimental Section)]. Finally, the salt diffusivity reduction factor featured in Table 1 was obtained via scaling the membrane salt permeability by the permeability the membrane would have with uncharged pores
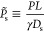
25where *D*_s_ is the bulk salt diffusion coefficient. We used the following bulk salt diffusion coefficients corresponding to the temperature of the measurements (296 K): *D*_KCl_ = 1.905 × 10^–9^ m^2^/s, and *D*_LiCl_ = 1.304 × 10^–9^ m^2^/s.

**Table 1:** Experimental Membrane Transport Properties and Fitted Surface-Charge Densities^a^

salt, concentrations	salt reflection coefficient (σ_s_)	salt diffusivity reduction (*P̃*_s_)	σ_s_/*P̃*_s_	surface-charge density (mC/m^2^)
KCl, 2 mM/1 mM	0.62	0.32	1.94	–5.7
KCl, 4 mM/2 mM	0.43	0.35	1.29	–9.5
LiCl, 2 mM/1 mM	0.53	0.48	1.10	–5.6
LiCl, 4 mM/2 mM	0.37	0.54	0.69	–9.3

a The space-charge model calculations were carried out for the average salt concentrations corresponding to the experimental conditions (1.5 and 3 mM) and for the two salts (KCl and LiCl) used in this study.

### Hydraulic Permeability and Pore Size

For the interpretation of osmotic pressure and diffusion permeance in terms of the capillary space-charge model, we need to know the pore size. Given that the membranes have identical straight cylindrical pores, this seems to be easy to obtain from the membrane hydraulic permeability by using the Hagen–Poiseuille equation. Actually, the situation is not that simple. The corresponding measurements and their interpretation are described in the Supporting Information. The pore radius was estimated to be 12 nm.

Table 1 shows a summary of the relevant experimental results and their fitting by the space-charge model.

Table 1 confirms the well-known trend, namely that the salt reflection coefficient in nanoporous charged membranes decreases with an increase in salt concentration. This occurs because the phenomenon is essentially controlled by the electrostatic exclusion of co-ions, which becomes weaker with an increase in concentration due to less EDL overlap. Salt diffusivity increases due to the same mechanism. In addition to this well-known trend, our results reveal that the surface-charge density considerably increases with salt concentration. Such an increase has already been reported before for this kind of membranes^22^ and was explained by a surface-charge regulation due to the increasing fraction of deprotonated (negatively charged) weakly acidic groups because of weakening electrostatic enhancement of the H^+^ concentration close to the pore surface. In nanopores whose radii are commensurate with the Debye screening length (the case in this study), this weakening is additionally enhanced by the decreasing degree of EDL overlap. This can explain the faster increase in surface-charge density than the square-root dependence on concentration predicted for both strongly overlapped and non-overlapped EDLs.^31,33^ Notably, the surface-charge density is practically independent of the kind of cation. This additionally confirms the simple mechanism of surface-charge formation due to deprotonation of a weakly acidic group without noticeable binding of single-charge cations of indifferent electrolytes.

Another nontrivial feature is the considerable dependence of both salt reflection coefficient and salt diffusivity reduction on the diffusion coefficient of the salt cation. At practically the same surface-charge density, both salt reflection and salt diffusivity reduction are less pronounced in LiCl than in KCl solutions. A decrease in the salt rejection with a decrease in diffusion coefficient of counterions has already been observed with TEMs in pressure-driven mode.^23^ This occurs because salt rejection is controlled not only by convective but also by electromigration transfer of co-ions across the membrane. The latter is proportional to the strength of the electric field arising due to the preferential transfer of counterions. This field is stronger for less mobile counterions owing to the lower conductivity of the pore solution.

## Conclusion

Parallel measurements of osmotic pressure and salt diffusion made possible elimination of the poorly controllable contribution of unstirred layers. Both of these phenomena in 24 nm charged nanopores could be quantitatively described by the classical space-charge model. The use of its full (numerical) version has eliminated uncertainties related to previous approximate solutions of the Poisson–Boltzmann equation. Experimental data obtained in dilute solutions of KCl and LiCl could be reproduced theoretically by using the surface-charge density as the only adjustable parameter. The observed concentration (and electrolyte-kind) dependencies of this density are in agreement with the model of deprotonation of weakly acidic groups on the pore walls without noticeable binding of counterions of dominant (1:1) electrolytes.

## Supporting Information Available

Derivation of transport equations, numerical procedures, contribution of volume transfer to salt diffusion, hydraulic permeability and pore size, membrane irradiation and etching procedures, experimental setup, and interpretation of diffusion measurements (PDF)

## Footnotes

a It is important to note that the ion convective flux in the pore is scaled on the ion concentration in the virtual solution.

b It is technically difficult to use other kind of stirrers in hydraulically closed compartments. This is explained in more detail in the Supporting Information.

c In our experimental setup, the half-cell volumes were slightly (∼20%) different, so we used the average value. The Supporting Information shows that the associated error is <1%.
