# Supplementary material for: Osmotic Pressure and Diffusion of Ions in Charged Nanopores
Source: Langmuir. 2021 Nov 25;37(48):14089–95. doi: 10.1021/acs.langmuir.1c02267 (PMC8656166; doi:10.1021/acs.langmuir.1c02267)
Supplement: Supplementary file 1 — la1c02267_si_001.pdf [file la1c02267_si_001.pdf]

Electronic Supporting Information to

Osmotic pressure and diffusion of ions in charged nanopores

P.Apel[a], M.Bondarenko[b], Yu.Yamauchi[a], A.Yaroshchuk[c,d*]

[a]Joint Institute for Nuclear Research, Joliot-Curie str. 6, 141980 Dubna, Russian Federation
[b]F.D.Ovcharenko Institute of Bio-Colloid Chemistry, National Academy of Sciences of Ukraine, Vernadskiy blvd.42, 03142, Kyiv, Ukraine
[c]ICREA, pg. L.Companys 23, 08010, Barcelona, Spain
[d]Department of Chemical Engineering, Polytechnic University of Catalonia, av. Diagonal 647, 08028, Barcelona, Spain

Derivation of transport equations.

In the system of coordinates fixed to the membrane matrix, we consider the local flux of an ion as a linear combination of electro-diffusion[1] (movement relative to the center of mass) and advection (movement with the center of mass).

$$\vec{j}_i = -\frac{D_i}{RT}\bar{c}_i \nabla \bar{\mu}_i^{(e)} + \bar{c}_i \vec{v} \qquad \text{(S1)}$$

where $\vec{j}_i$ is the local flux of ion "i", D_i is the ion diffusion coefficient, R is the universal gas constant, T is the absolute temperature, $\bar{c}_i$ is the local ion concentration, $\bar{\mu}_i^{(e)}$ is the ion electrochemical potential, $\vec{v}$ is the local fluid velocity.

The fluid movement can be a combination of pressure-driven flow and (electro)osmosis and is controlled by external perturbations (for example, a hydrostatic pressure difference) as well as by ion distribution inside pores. Within the scope of standard space-charge model used in this study, the flow dynamics is described by Stokes equation including a body force, $\vec{f}$, arising due to space charges near pore surfaces and induced transmembrane electric fields.

$$\eta \nabla^2 \vec{v} = \nabla \bar{p} - \vec{f} \qquad \text{(S2)}$$

where η is the dynamic viscosity, $\bar{p}$ is the hydrostatic pressure inside the pore. This body force is equal to the negative gradient of electrostatic potential times local electric space-charge density, which can be expressed through local ion concentrations

$$\vec{f} = -F \nabla \bar{\varphi} \sum_i Z_i \bar{c}_i \qquad \text{(S3)}$$

where the summation extends over all the ions. By using this definition of electrochemical potential

[1] Although no external voltage is applied in this study, there are spontaneously-arising electric fields inside the membrane nanopores.

$$\nabla\bar{\mu}_i^{(e)} \equiv \nabla\bar{\mu}_i^{(c)} + FZ_i\bar{\varphi} \quad \text{(S4)}$$

we can express the gradient of electrostatic potential via gradients of electrochemical and chemical potentials to obtain for the body force

$$\vec{f} = -\sum_i \bar{c}_i\left(\nabla\bar{\mu}_i^{(e)} - \nabla\bar{\mu}_i^{(c)}\right) \equiv -\sum_i \bar{c}_i \nabla\bar{\mu}_i^{(e)} + \nabla\bar{\Pi} \quad \text{(S5)}$$

where $\bar{\Pi} \equiv \sum_i \bar{c}_i \bar{\mu}_i^{(c)}$ is the local osmotic pressure inside pores. Consequently, Stokes equation Eq(S2) can be transformed this way

$$\eta\nabla^2\vec{v} = \nabla(\bar{p} - \bar{\Pi}) + \sum_i \bar{c}_i \nabla\bar{\mu}_i^{(e)} \quad \text{(S6)}$$

The first term is proportional to the negative gradient of solvent chemical potential defined as

$$\bar{\mu}_{solv} \equiv \bar{V}_{sol}(\bar{p} - \bar{\Pi}) \quad \text{(S7)}$$

where $\bar{V}_{sol}$ is the partial molar volume of solvent. Eq(S6) is convenient for a local-equilibrium analysis because the gradients of (electro)chemical potentials can be considered to be the same as in a virtual bulk electrolyte solution that could be in thermodynamic equilibrium with a given cross-section inside a pore. Assuming that the pores are sufficiently small, these gradients are independent of the position inside the pore. The gradient of chemical potential of solvent in the virtual solution is also the same as in the pore. Additionally, in this study we assume that the partial molar volume of solvent in the pores is the same as in the virtual solution (no changes in the solvent properties in nanopores), so its partitioning coefficient is equal to one, and

$$\nabla(\bar{p} - \bar{\Pi}) = \nabla(p - \Pi) \quad \text{(S8)}$$

where the properties without bar correspond to the virtual solution. The local ion concentrations inside pores can be related to the concentrations in the virtual solution, c_i, via ion partitioning coefficients, Γ_i,

$$\bar{c}_i = c_i\Gamma_i \quad \text{(S9)}$$

Thus, the Stokes equation can be written down in this form where its right-hand side contains only gradients in the virtual solution.

$$\eta\nabla^2\vec{v} = \nabla(p - \Pi) + \sum_{i=1}^{n} \Gamma_i c_i \nabla\mu_i^{(e)} \quad \text{(S10)}$$

Following [1] we introduce a linear functional operator, $\hat{F}[]$ giving a solution to this equation

$$\eta\nabla^2\vec{v} = -\vec{g} \quad \text{(S11)}$$

where $\vec{g}$ is an arbitrary function of coordinate inside the pore. Given that neither the gradients nor the ion concentrations in the virtual solution depend on this coordinate, they can be taken out of the operator sign, so

$$\vec{v} = -\hat{F}[1]\cdot\nabla(p - \Pi) - \sum_{i=1}^{n} \hat{F}[\Gamma_i]\cdot c_i \cdot \nabla\mu_i^{(e)} \quad \text{(S12)}$$

The form of operator $\hat{F}[]$ depends on the pore geometry. In this study, in agreement with the experimental part using track-etched membranes, we will further consider long straight cylindrical pores of equal size. In this case, all the flows are 1D, so we will further drop the

vector signs. Besides, the ion distribution coefficients, Γ_i, depend only on the radial coordinate inside the pore. The operator can be shown to have this form [2]

$$\hat{F}[\Gamma_i] = -\frac{r_p^2}{\eta}\left[ln(\rho)\int_0^\rho d\rho'\rho'\Gamma_i(\rho') + \int_\rho^1 d\rho'\rho' ln(\rho')\Gamma_i(\rho')\right] \quad \text{(S13)}$$

where r_p is the pore radius, $\rho \equiv {}^{r}/_{r_p}$ is the dimensionless radial coordinate. For a given ion (after multiplication by the negative gradient of the corresponding electrochemical potential), this gives the "iono-osmotic" velocity profile. For the solvent ($\Gamma_{solv} = 1$), we obtain the well-known parabolic profile

$$\hat{F}[1] = \frac{r_p^2}{4\eta}(1-\rho^2) \quad \text{(S14)}$$

To obtain observable ion fluxes, we should substitute Eq(S12) into Eq(S1) (applying the concept of local equilibrium to the first term and to the ion concentrations in the second) and average over the pore cross-section. As a result, for solutions of single salts (two ions), we obtain

$$-J_1 = c_1 \cdot \left[\langle\Gamma_1\hat{F}[1]\rangle\nabla p + c_1\langle\Gamma_1\hat{F}[\Gamma_1 - 1]\rangle\nabla\mu_1^{(e)} + c_2\langle\Gamma_1\hat{F}[\Gamma_2 - 1]\rangle\nabla\mu_2^{(e)} + \frac{\langle D_1\Gamma_1\rangle}{RT}\nabla\mu_1^{(e)}\right] \quad \text{(S15)}$$

$$-J_2 = c_2 \cdot \left[\langle\Gamma_2\hat{F}[1]\rangle\nabla p + c_2\langle\Gamma_2\hat{F}[\Gamma_2 - 1]\rangle\nabla\mu_2^{(e)} + c_1\langle\Gamma_2\hat{F}[\Gamma_1 - 1]\rangle\nabla\mu_1^{(e)} + \frac{\langle D_2\Gamma_2\rangle}{RT}\nabla\mu_2^{(e)}\right] \quad \text{(S16)}$$

where $J_i \equiv \langle j_i\rangle$, the brackets, $\langle\rangle$, mean averaging over the pore cross-section, that is integration over the radial coordinate times 2π, and scaling on the area of pore cross-section. The solvent flux (considered to be equal to the volume flux in the approximation of dilute solutions) is obtained via averaging of Eq(S12).

$$-J_v = \langle\hat{F}[1]\rangle\nabla p + c_1\langle\hat{F}[\Gamma_1 - 1]\rangle\nabla\mu_1^{(e)} + c_2\langle\hat{F}[\Gamma_2 - 1]\rangle\nabla\mu_2^{(e)} \quad \text{(S17)}$$

For the cylindrical capillary model,

$$\langle\Gamma_i F[\Gamma_j]\rangle \equiv -\frac{2r_p^2}{\eta}\int_0^1 d\rho\rho\Gamma_i(\rho)\left[ln(\rho)\int_0^\rho d\rho'\rho'\Gamma_j(\rho') + \int_\rho^1 d\rho'\rho' ln(\rho')\Gamma_j(\rho')\right] \quad \text{(S18)}$$

Now, we will transform Eqs(S15,S16) to have electric-current density and transmembrane volume flow singled out explicitly. This will enable us to define membrane transport processes (in particular, salt diffusion flux) at zero current and volume flow as occurs in the measurements of osmotic pressure. For this, we express the gradient of virtual hydrostatic pressure via the volume flux and gradients of electrochemical potentials by using Eq(19) and substitute to Eqs(S15,S16). After some identical transformation, we obtain

$$J_1 = -c_1 \cdot \left[\frac{1}{RT}\left(P_1\nabla\mu_1^{(e)} + \frac{\omega}{\nu_1}\nabla\mu_2^{(e)}\right) - J_v\tau_1\right] \quad \text{(S19)}$$

$$J_2 = -c_2 \cdot \left[\frac{1}{RT}\left(P_2\nabla\mu_2^{(e)} + \frac{\omega}{\nu_2}\nabla\mu_1^{(e)}\right) - J_v\tau_2\right] \quad \text{(S20)}$$

$$\nabla p = -\frac{J_v}{\langle F[1]\rangle} + c_1(1-\tau_1)\nabla\mu_1^{(e)} + c_2(1-\tau_2)\nabla\mu_2^{(e)} \quad \text{(S21)}$$

where we have denoted

$$P_i \equiv \langle D_i\Gamma_i\rangle + RTc_i\langle F[1]\rangle\left(\frac{\langle\Gamma_i F[\Gamma_i]\rangle}{\langle F[1]\rangle} - \tau_i^2\right) \quad \text{(S22)}$$

$$\omega \equiv RT\nu_1\nu_2 c\langle F[1]\rangle \cdot \left(\frac{\langle \Gamma_1 F[\Gamma_2]\rangle}{\langle F[1]\rangle} - \tau_1\tau_2\right) \quad \text{(S23)}$$

$$\tau_i \equiv \frac{\langle \Gamma_i F[1]\rangle}{\langle F[1]\rangle} \quad \text{(S24)}$$

c is the electrolyte concentration, $c_i \equiv \nu_i c$, ν_i are ion stoichiometric coefficients, they satisfy electroneutrality condition, $Z_1\nu_1 + Z_2\nu_2 = 0$. The ion transmission coefficients, τ_i, quantify the extent to which ions are convectively entrained by the volume flow[2]. Notably, they are larger than one for counterions whose partitioning coefficients exceed unity. In principle, these coefficients can be affected by steric hindrance[3] but this is not significant in nanopores whose size is much larger than the ion size (the focus of this study). Based on the same considerations, we also neglect the effect of steric hindrance on the ion diffusion and consider ion diffusion coefficients in nanopores constant and equal to those in bulk electrolyte solution.

Electrochemical potentials of ions are difficult to control. Therefore, it is convenient to transform Eqs(S19-S21) in a form containing more manageable quantities such as electrostatic-potential and salt-concentration gradients as well as electric-current density and salt flux. The electric-current density is defined this way

$$I \equiv Z_1 J_1 + Z_2 J_2 \quad \text{(S25)}$$

The definition of salt flux is less trivial because at non-zero electric currents changes in the salt concentration in reservoirs separated by a membrane are affected by the ion generation or consumption at electrodes (or by ion fluxes through other membranes like in electrodialysis). In our particular experimental conditions, the electric-current density is zero, so this issue does not arise. We use this symmetrical definition of salt flux

$$J_s \equiv \frac{1}{2}\left(J_1/\nu_1 + J_2/\nu_2\right) \quad \text{(S26)}$$

This expression for the gradients of electrochemical potential[3]

$$\nabla\mu_{1,2}^{(e)} \equiv RT\frac{\nabla c}{c} + FZ_{1,2}\nabla\varphi \quad \text{(S27)}$$

is substituted to Eqs(S19-S21) and ion fluxes are expressed via current density and salt flux. After some identical transformations, we obtain:

$$J_v\left(\frac{1}{\chi} + \frac{\rho_{ek}^2}{g}\right) = -\nabla p + \frac{\rho_{ek}}{g} I + RT(\nu_1 + \nu_2)(1 - \mathrm{T}_s) \cdot \nabla c \quad \text{(S28)}$$

$$J_s = -P_s\nabla c + \frac{I \cdot (t_1 - t_2)}{2F(Z_1\nu_1)} + J_v c\mathrm{T}_s \quad \text{(S29)}$$

$$-\nabla\varphi = \frac{I - \rho_{ek} \cdot J_v}{g} + \frac{RT}{F}\left(\frac{t_1}{Z_1} + \frac{t_2}{Z_2}\right)\frac{\nabla c}{c} \quad \text{(S30)}$$

$$\chi \equiv \langle F[1]\rangle = \frac{r_p^2}{8\eta} \quad \text{(S31)}$$

---

[2] It is important to note that the ion convective flux in the pore is scaled on the ion concentration in the virtual solution.

[3] From now on, we assume the solution to be ideal.

χ is the hydraulic permeability at zero voltage gradient. Eq(S28) shows that the more easily measurable hydraulic permeability at zero electric current is equal to

$$\chi_* \equiv \frac{\chi}{1 + \chi \cdot \frac{\rho_{ek}^2}{g}} \tag{S32}$$

$$\rho_{ek} \equiv F(Z_1 \nu_1) \cdot c \cdot (\tau_1 - \tau_2) \tag{S33}$$

is the electrokinetic charge density (the proportionality coefficient between electric-current density and volume flux under streaming-current conditions, i.e. $\nabla c = 0, \nabla \varphi = 0$)

$$g \equiv \frac{F^2}{RT}(Z_1 \nu_1) c \left[Z_1 \left(P_1 - \frac{\omega}{\nu_2} \right) - Z_2 \left(P_2 - \frac{\omega}{\nu_1} \right) \right] \tag{S34}$$

is the electric conductivity at zero transmembrane volume flow,

$$P_s \equiv \frac{(Z_1 - Z_2)\left(P_1 P_2 - \frac{\omega^2}{\nu_1 \nu_2} \right)}{Z_1 \left(P_1 - \frac{\omega}{\nu_2} \right) - Z_2 \left(P_2 - \frac{\omega}{\nu_1} \right)} \tag{S35}$$

is the salt diffusion permeability at zero transmembrane volume flow,

$$\mathrm{T}_s \equiv \tau_1 t_2 + \tau_2 t_1 \tag{S36}$$

is the salt transmission coefficient (one minus salt reflection coefficient),

$$t_1 \equiv \frac{Z_1 \left(P_1 - \frac{\omega}{\nu_2} \right)}{Z_1 \left(P_1 - \frac{\omega}{\nu_2} \right) - Z_2 \left(P_2 - \frac{\omega}{\nu_1} \right)} \tag{S37}$$

is the transport number of ion “1” at zero transmembrane volume flow.

$$t_2 \equiv 1 - t_1 \tag{S38}$$

by definition. The coefficients in Eqs(S28-S37) are given by Eqs(S22-S24). By putting $I = 0$ from Eqs(S28, S29), we obtain the starting Eqs(1,2) of the main text.

Numerical procedures

Non-linearized Poisson-Boltzmann equation was solved numerically by using 6th-order Runge-Kutta routine. Electrostatic potential on the capillary axis was iterated until the boundary condition for the potential derivative at the capillary wall (Eq(16)) was satisfied. The integrals from Eqs(8-10,12) were calculated numerically by using the method of trapezoids. The overall numerical error was less than 0.1%. Numerical procedures were implemented using Delphi 2017.01 software.

Contribution of volume transfer to salt diffusion

In experiments, salt diffusion permeability was estimated from the rate of change of salt concentration difference between the source and the receiving compartments. In our experiments, the relative concentration-difference changes were rather small (mostly because the measurements were performed at relatively small concentration differences, so the receiving concentration could not be very low and rather small relatively changes in the concentration had to be detected), so the accuracy of salt-flux estimates is not very high. Accordingly, it is difficult to differentiate between the rate of salt flux occurring at initial stages

of experiment (where the osmotic flow is still non-zero, see Fig.1a) and at the later stages where the salt diffusion occurs at practically zero transmembrane volume flow. Nonetheless, the theoretical model developed above affords estimates of the possible contribution of volume transfer to salt diffusion (and demonstration that it is moderate, see below). While defining the diffusion permeability at non-zero volume flow we should account for the concentration changes due to solvent transfer from the receiving compartment. This means that we should consider the so-called salt chemical flux defined as

$$J_s^{(ch)} \equiv J_s - cJ_v \qquad \text{(S39)}$$

and quantifying the rate of salt-concentration changes in the compartment receiving salt flux. In our experiments, the contribution of volume transfer to the salt diffusion is largest at the early stages where the transmembrane pressure difference is still very low, so for overestimates we can assume it to be equal to zero. By putting $\nabla p = 0$, $I = 0$ in Eq(S28) and by substituting the resulting expression for the osmotic volume flux to Eqs(S29, S39), we obtain

$$J_s^{(ch)} = -\left[P_s + RTc(\nu_1 + \nu_2)\chi_* \sigma_s^2\right]\nabla c \qquad \text{(S40)}$$

Accordingly, the "initial" salt diffusion permeability (at zero hydrostatic-pressure difference) is related to the "zero-flow" permeability this way.

$$P_s|_{\Delta P=0} = P_s|_{J_v=0} + RT(\nu_1 + \nu_2)c\chi_* \sigma_s^2 \qquad \text{(S41)}$$

Since all the factors in the second term in the right-hand side of Eq(48) are positive, due to volume transfer, salt diffusion permeability always increases.

At non-zero transmembrane volume flows, the increased rate of salt-concentration changes in the salt-receiving compartment is due to the (partial) salt rejection accompanying the osmotic flow leaving this compartment. Since both the rate of osmosis and the salt rejection are proportional to the salt reflection coefficient, the correction is quadratic in it. As we can see from Eq(S41), the osmotic correction to the membrane diffusion permeability can be expected to be noticeable just for the investigated "intermediate" nanoporous charged membranes because such membranes can have relatively large hydraulic permeabilities along with not too small salt reflection coefficients at not too low salt concentrations. True, the effect is still limited because "large" salt-reflection coefficients ($\sigma_s \approx 1$) occur only in quite dilute solutions (note the proportionality of the correction to the virtual salt concentration). Fig.S1 shows the results of some calculations of this correction for parameter combinations corresponding to the membranes and conditions described in the experimental section. The values of surface-charge density are close to those fitted to the experimental data.

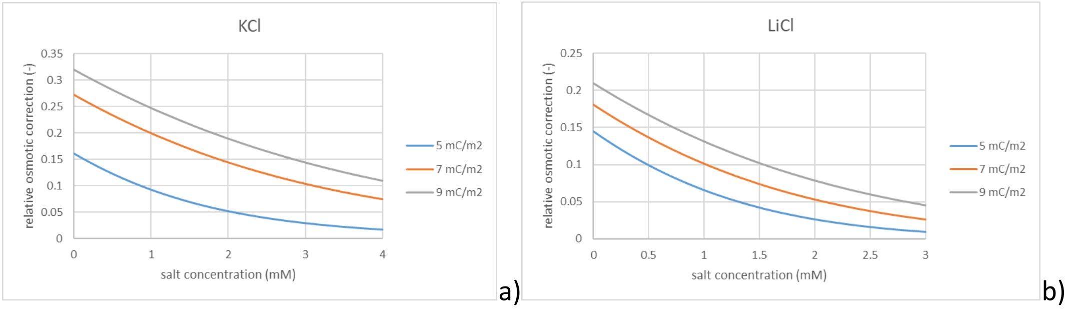

Fig.S1. Relative correction to salt permeability due to volume transfer (theoretical)

In very dilute solutions and at larger surface-charge densities, the correction is significant (especially, in the case of KCl). However, in the investigated membranes the surface-charge density noticeably decreases with the salt concentration (see the main text). Thus for instance, at average KCl concentration of 1.5 mM the surface-charge density is about -5.7 mC/m^2. Fig.S1a) shows that the relative osmotic correction in this case is below 10%. At the higher average concentration of 3 mM KCl, the fitted surface-charge density is about -9.5 mC/m^2. In this case, the correction is around 15%. For LiCl, the corrections are smaller. We should also keep in mind that Fig.S1 shows the largest corrections occurring at strictly vanishing hydrostatic-pressure difference while in our experiments this condition is fulfilled only at very early stages, while the system progressively approaches the mode of zero volume flow with time. Given the relatively low accuracy of determination of rate of change of salt concentration (see above) we will neglect this relatively small correction and use the zero-flow diffusion permeability (Eq(37)) for the fitting of experimental data.

Hydraulic permeability and pore size

For the interpretation of osmotic pressure and diffusion permeance in terms of capillary space-charge model, we need to know the pore size. Given that the membranes have identical straight cylindrical pores this seems to be easy to obtain from the membrane hydraulic permeability by using Hagen-Poiseuille equation. Keeping in mind the salt-concentration dependence of hydraulic permeability (due to the so-called electro-viscosity, see Eq(S28)) at first glance it appears logical to make this measurement using electrolyte solutions of concentrations corresponding to those employed in the other measurements. However, this seemingly simple measurement is actually non-trivial in interpretation primarily due to a salt rejection and the corresponding buildup of osmotic-pressure difference. In terms of trans-membrane volume flow vs applied hydrostatic pressure, this leads to a characteristic dependence having an initial and a subsequent linear parts, the extension of the latter crossing the pressure axis at a non-zero positive pressure (see, for example, Fig.2 of ref.[3]). Determination of the genuine hydraulic permeance requires reaching well into this second linear part (to be able to determine its slope). For solutions of concentrations used in the principal measurements (1-4 mM), the corresponding pressures cannot be reached in our setup. Besides, such interpretation requires a careful control of stirring conditions, which is also not feasible in stirred test cells. Therefore, for the measurements of hydraulic permeability, we opted for the use of pure water. True, the contribution of electro-viscosity correction in this case can be different from solutions of finite electrolyte concentrations. However, given the strong (fourth-power) dependence of hydraulic permeability on the pore size and the limited magnitude of electro-viscous phenomenon (max. ca.25% in KCl and ca.40% in LiCl solutions[1]) the associated error in the determination of the pore size can only be moderate. Besides, below we will see that surface-charge density in the investigated membranes noticeably decreases with electrolyte concentration. If we extrapolate this trend to very dilute solutions, the electro-viscosity correction (controlled in very dilute solutions by the surface-charge density[1]) can be expected to be negligible, and the especially simple Eq(S31) to be applicable for the determination of pore size.

Membrane irradiation and etching procedures

The angle distribution of pore orientation with respect to the membrane surface originates from the irradiation mode illustrated by Fig.S2. The film circumflexes a horizontal cylindrical shaft 4 cm in radius. The ion beam, homogeneously spread in both vertical and horizontal

directions, impinges the film through a window 4 cm high. With such geometry, all orientations within the angle range from -30 to +30 degrees are equally likely.

Due to the pore "non-perpendicularity" the pore crossing occurred only at some punctual positions along the pore length. Such events give rise to some deviations from the model geometry but the most important parameter, namely, the pore size is only slightly affected. An alternative scenario of track-etched membrane with parallel pores would be a much worse model system. The pores in TEMs are distributed over the surface stochastically. Some of the pores form double, triple and so on clusters. Several clusters are seen in Fig. 1 (right). When the pore channels are parallel, each cluster is a channel with a larger (compared to singles) cross-section. The number of such clusters can be calculated[4]. At a pore density of 8E13 m^{-2}, there are plenty of multiples in a sample 2 cm in diameter. The multiples strongly affect the membrane selectivity. The angle distribution of pore axes (± 30 degrees) reduces the number of multiples by several orders of magnitude.

The membranes were produced in the following way. PET film 10 um thick was irradiated with 1 MeV/u Xe ions, then treated with soft UV radiation to sensitize tracks and etched in 0.5 M NaOH at 80°C for 6.2 min. Under such conditions, the track cores etched though for the time shorter than 1 min. The rest of time (1-6 min) corresponds to a slow widening of pores. Since the total etching time is at least 6 times longer than the breakthrough time, the pore channels should be cylindrical. No narrowing of the channels in the middle of the film is seen in the SEM images of cross sections of the membrane (Fig. 1, left).

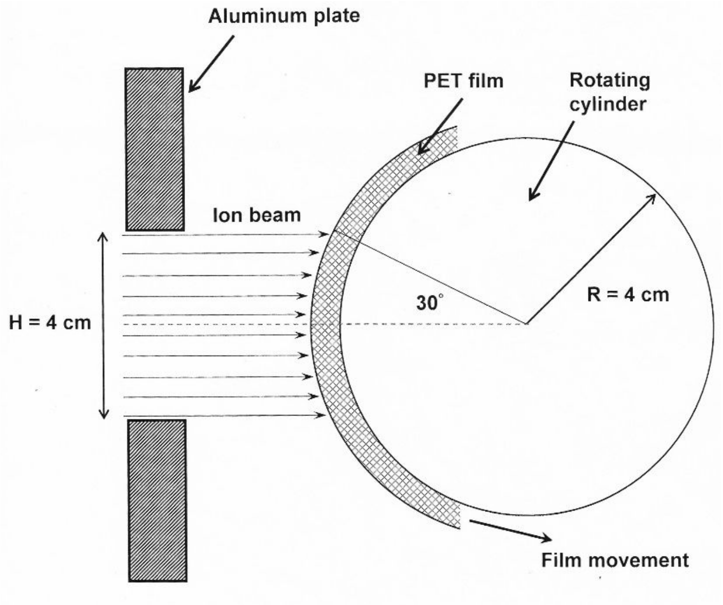

Fig.S2. Schematic of film irradiation

Experimental setup

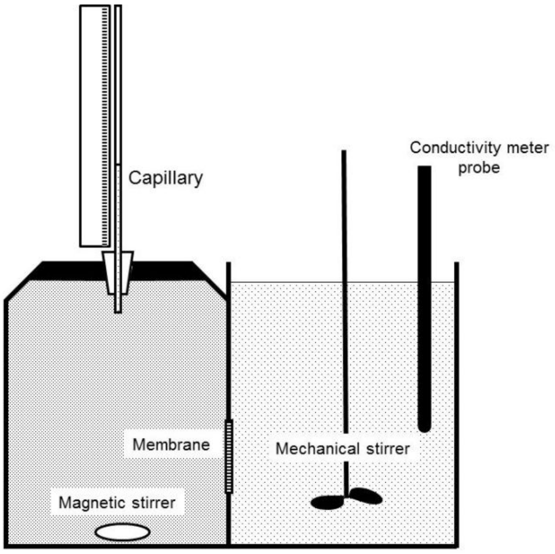

Fig.S3 Schematic of experimental setup

To avoid leaks of solution in the left-hand compartment we used a magnetic stirrer (even a tiny leak affects the results). The right-hand compartment is open and, therefore, it was possible to use a "pony mixer", which is more effective and easier to control.

Interpretation of diffusion measurements

The maximum rise of solution level in the measuring capillary in our measurements did not exceed ca.35 cm. Given the inner diameter of the capillary equal to 1 mm, the corresponding change of volume was about 3 ml, that is ca.1% of the compartment volumes. Therefore, in the following, we will neglect the contribution of volume transfer to the changes of solute concentrations. In this approximation, from the material balances we can obtain:

$$-V_1\frac{dc_1}{dt} = P \cdot A \cdot (c_1 - c_2) \qquad \text{(S42)}$$

$$V_2\frac{dc_2}{dt} = P \cdot A \cdot (c_1 - c_2) \qquad \text{(S43)}$$

$$V_1c_1 + V_2c_2 = Q \qquad \text{(S44)}$$

where Q is a constant total amount of salt, P is the membrane diffusion permeance, A is the membrane area, V_1, V_2 and c_1, c_2 are the compartment volumes (assumed to be constant), and (time-dependent) solute concentrations in them. By introducing average compartment volume, $\overline{V} \equiv \frac{V_1 + V_2}{2}$, and the relative deviation from the average, $\Delta V \equiv \frac{V_1 - V_2}{2}$, Eqs(S42,S43) can be transformed to

$$-\frac{d}{dt}[\overline{V}(c_1 - c_2) + \Delta V(c_1 + c_2)] = 2P \cdot A \cdot (c_1 - c_2) \qquad \text{(S45)}$$

Eq(S44) can be used to express the time derivative of the sum of concentrations via time derivative of their difference, so solving the simple resulting ODE we obtain

$$ln\left(\frac{\Delta c(t)}{\Delta c(0)}\right) = -\frac{2PA}{\overline{V} - (\Delta V/\overline{V})^2} \cdot t \qquad \text{(S46)}$$

One can see that the correction due to different compartment volumes is quadratic in $(\Delta V/\overline{V})$, so for our volumes (250 ml and 300 ml) it is about 0.8% and can be neglected.

(1) Yaroshchuk, A. E. Osmosis and Reverse Osmosis in Fine-Porous Charged Diaphragms and Membranes. *Adv. Colloid Interface Sci.* **1995**, *60*, 1–93.

(2) Yaroshchuk, A.; Bondarenko, M. P. M. P. Current-Induced Concentration Polarization of Nanoporous Media: Role of Electroosmosis. *Small* **2018**, *14* (18), 1703723. https://doi.org/10.1002/SMLL.201703723.

(3) Yaroshchuk, A.; Bruening, M. L.; Zholkovskiy, E. Modelling Nanofiltration of Electrolyte Solutions. *Adv. Colloid Interface Sci.* **2019**, *268*. https://doi.org/10.1016/j.cis.2019.03.004.

(4) Riedel, C.; Spohr, R. Correcting Overlapping Counts in Dose Calibration at High Event-Densities. *Nucl. Tracks* **1981**, *5* (3), 265–270. https://doi.org/10.1016/0191-278X(81)90004-4.
